# Comparison of Lifestyle Factors, Dietary Intake, and Physical Activity Among Female Chinese University Students With and Without Normal Weight Obesity

**DOI:** 10.7759/cureus.114154

**Published:** 2026-08-08

**Authors:** Miho Sugiura, Shian-Yang Tzeng, Minato Nakazawa

**Affiliations:** 1 Public Health, Kobe University Graduate School of Health Sciences, Kobe, JPN; 2 School of Economics and Management, Quanzhou University of Information Engineering, Quanzhou, CHN

**Keywords:** body fat percentage, dietary records, lifestyle factors, normal weight obesity, physical activity, snack consumption

## Abstract

Aims

Normal weight obesity (NWO), which refers to individuals with a normal body mass index (BMI) but an excessive body fat percentage (BF%), is associated with metabolic disorders. Despite its high prevalence among Chinese female university students, research on lifestyle, particularly diet, in this population remains limited. We investigated any differences in these factors between students categorized as NWO and non-NWO.

Methods

The cross-sectional study comprised 70 female university students aged 18-23 years. Participants were classified as NWO (BMI: 18.5-23.9 kg/m², BF%: ≥20% in male individuals, ≥30% in female individuals) or non-NWO (BMI: 18.5-23.9 kg/m², BF%: <20% in male individuals, <30% in female individuals). Anthropometric measurements, dietary intake (via dietary records), and physical activity were assessed. Dietary intake assessment utilized food photographs, weighed portions, and detailed ingredient tracking. Nutrient composition was analyzed using the Chinese and Japanese food composition tables. Statistical analyses compared dietary composition, physical activity, and lifestyle behaviors between groups.

Results

Total energy intake did not differ between the groups. However, snack calorie intake differed between the NWO and non-NWO groups. Additionally, snack calorie ratio (%) tended to differ between them. Both groups had comparable total physical activity and sedentary time.

Conclusion

Snack volume and pattern of consumption should be addressed to prevent NWO among Chinese female university students.

## Introduction

Obesity represents a major public health problem, with global prevalence tripling over the past five decades [1]. Between 2004 and 2018 in China, the number of obese individuals increased threefold, reaching approximately 85 million, representing 8.5% of the population [2]. Consequently, obesity-related complications, such as liver disease, are prevalent in China [3]. Moreover, a significant proportion of school-aged children and adolescents are affected, and obesity often persists into adulthood [4]. Obesity is typically evaluated using body mass index (BMI), classifying individuals with a BMI ≥25 as overweight and those with a BMI ≥30 as obese [5]. However, BMI does not account for fat mass or its distribution and inaccurately classifies approximately 30% of people with excessive body fat percentage (BF%) [6]. Screening for adiposity in individuals with a normal BMI can help identify those at increased risk for cardiometabolic disturbances and cardiovascular disease-related mortality [7]. Elevated fat mass, rather than weight or BMI, is a significant risk factor for metabolic diseases [8]. The recently defined normal weight obesity (NWO) phenotype describes individuals with a normal BMI and elevated BF% (male individuals: ≥20% and female individuals: ≥30%) [9]. Some evidence suggests that individuals with NWO exhibit a fourfold higher prevalence of metabolic syndrome than do those without NWO; meanwhile, female individuals with NWO also experience more than twice the risk of cardiovascular mortality compared to that experienced by those without NWO [10]. Therefore, relying solely on BMI to assess obesity may limit efforts to prevent chronic diseases and overlook necessary interventions for individuals with NWO. This limitation is particularly relevant in China, which faces a substantial and rapidly increasing obese population, including a high obesity prevalence among young people. Several studies have indicated that lifestyle factors such as diet and physical activity contribute to NWO incidence in young people. Individuals with NWO exhibit lower physical strength and reduced physical activity levels [11-13]. Meanwhile, little is known about the associated dietary patterns. A Finnish study found no association between NWO and energy intake in adults [14], a result consistent with that of an analysis of data from 18-year-olds in Iceland [15]. Similarly, a study of American adults reported that dietary quality did not differ between individuals with and without NWO [16]. However, a study in Iran found that male university students with NWO, similar to those with obesity, exhibited higher energy intake than did their peers with normal weight [17]. Furthermore, NWO status has been associated with low vegetable consumption and high intake of unhealthy foods [14,15,18]. Additionally, most prior studies have relied on food frequency questionnaires and 24-hour recall, which may compromise the accuracy of nutrient composition estimates because of their inherent limitations. Dietary records are widely recognized as the most accurate method for estimating actual diet and nutrient intake [19], as they involve prospective, open-ended data collection over a specified period.

Therefore, the aim of this study was to compare lifestyle factors, dietary intake, and physical activity between female Chinese university students with NWO and those without NWO.

## Materials and methods

This cross-sectional study recruited 177 university students aged 18 to 23 years from the Sanshui campus of Guangdong University of Finance and Economics, China, using convenience sampling between April 15 and June 25, 2023.

The study recruited healthy undergraduate and postgraduate students enrolled at the university. Many of the subjects were freshmen and second-year students, and 99% of the participants lived in the provided university accommodations. Most were permanent residents of Guangdong Province in southern coastal China, a populous and economically prosperous region. Individuals diagnosed with chronic diseases, endocrine disorders, including diabetes, or those receiving long-term oral medications were excluded.

Exclusion of cases with missing data reduced the sample size from 177 to 150. Subsequent anthropometric assessments using NWO and non-NWO criteria further decreased the sample to 88 participants. Although a high body fat percentage (BF%) was observed in two male participants (6%) and 34 female participants (29%) among the 150 participants, individuals classified as underweight, overweight, or obese with high BF% were excluded from the analysis. As no male participants met the NWO criteria, all male participants were excluded, resulting in a final sample of 70 female participants (24 with NWO and 46 with non-NWO). Consequently, approximately 60% of students were excluded for failing to meet the eligibility criteria. 

Given the lack of difference and standard deviation values reported in prior studies, which are necessary for calculations, the sample size was determined based on statistical analysis conventions applicable to multiple regression models [20]. Participants were recruited on campus near the west entrance, where trained research assistants (RAs) explained the study’s purpose and procedures using an informational leaflet. Participation was voluntary. After agreeing to take part, participants accessed WPS online files to provide informed consent and complete questionnaire surveys. Dietary records were collected with assistance from a dietitian and trained RAs, while anthropometric measurements were collected in a classroom setting. 

**Figure 1:**
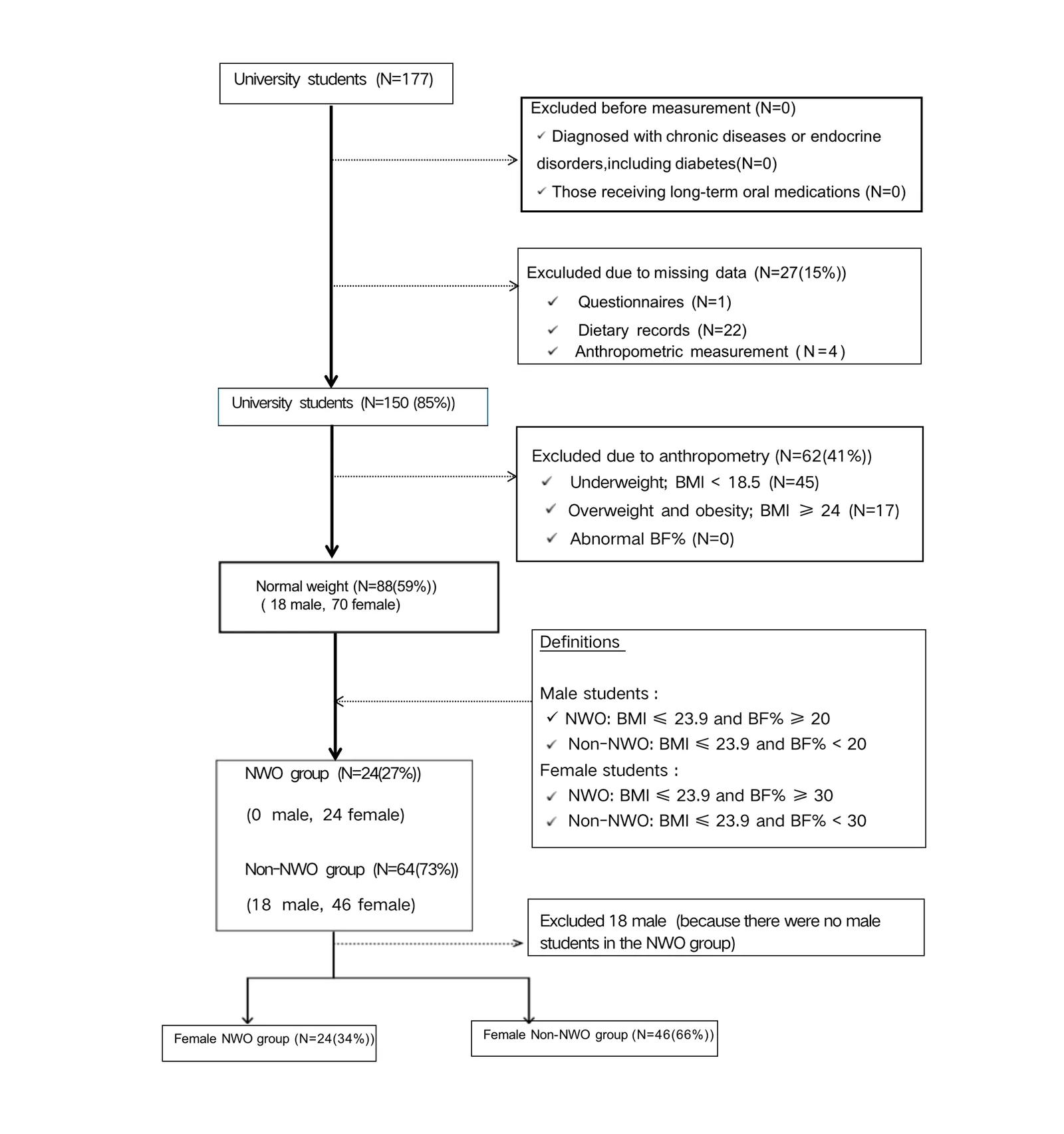
Flowchart of study participant selection NWO, normal-weight obesity; BMI, body mass index; BF%, body fat percentage

NWO was defined according to the criteria established by the Working Group on Obesity in China [21]. Underweight was classified as BMI <18.5, overweight as BMI ≥24, and obesity as BMI ≥28. Participants with a normal BMI (18.5 ≤ BMI ≤ 23.9) but excessive BF% (male participants: ≥20%, female participants: ≥30%) were categorized as NWO, whereas those with a normal BMI and low BF% (male participants: <20%, female participants: <30%) were classified as non-NWO [15]. Figure 1 illustrates the participant flowchart.

Dietary records

Dietary records are a subjective dietary assessment method that evaluates actual food intake over a specific period using self-administered questionnaires [22]. The advantages of dietary records include a lower likelihood of omitting or forgetting food items and eliminating reliance on portion size estimation, as exact portion sizes are recorded [19]. Dietary intake was assessed using dietary records detailing mealtimes, food sources, food names, ingredients, quantities, and accompanying photographs. A standardized food measurement flowchart was provided to ensure accuracy. Participants were instructed to photograph their entire meal, including any nutrition composition labels if available; weigh and photograph individual dishes, displaying the weight on a scale; weigh and photograph any leftover food; and record the food name and manually calculate the actual intake.

Key guidelines included maintaining usual eating habits, measuring food before or during meals, and promptly recording intake instead of waiting until the last day. If sharing food, participants were advised to separate their portion at the outset and note the number of individuals sharing. The meal format (self-cooked or served) was also recorded, as cooking methods influence nutritional composition.

Over three semesters, RAs participated in more than 20 training sessions focused on data collection procedures, cooking and food weighing techniques, and calculations of nutritional values. The training emphasized the study objectives, data collection methods, and instructions for completing food record sheets, including the measurement of cooked food and the use of a standardized kitchen scale. This preparation enabled RAs to effectively assist participants who were uncertain about recording meals or using the scale. Given that 99% of university students reside in university dormitories during the semester, the primary sources of food were campus cafeterias and nearby restaurants.

Dietary intake was measured in three sequential steps: (i) identification of food and beverage types and categories, (ii) documentation of nutritional content based on nutritional labeling or by weighing the food and beverage items, and (iii) calculating the nutritional value of the collected food records.

First, participants were instructed to complete detailed records of their food sources. To facilitate accurate reporting and minimize errors or confusion in dish nomenclature, the research team compiled a comprehensive list of university canteens, on-campus restaurants, and surrounding off-campus establishments, along with their respective menus, for participant selection. For food sources not listed, participants manually entered the details.

Second, all participants were provided with identical model electronic scales to record the net weight of all dietary items consumed over three consecutive days. A standardized weighing protocol was developed and disseminated to RAs, ensuring uniform instruction for participants on measuring net weights. For packaged foods, the net weight indicated on the packaging was used; participants were required to deduct the weight of uneaten portions from both packaged and unpackaged items, either via electronic scale (when feasible) or by self-estimated percentage for items such as partially consumed beverages. Following weight/proportion recording, participants photographed their meals before and after consumption (if not fully eaten) to enable subsequent verification and caloric computation by RAs. Given the characteristics of Chinese cuisine, certain foods involved participant selection of raw ingredients (e.g., “ma-la-tang” hot pot), which were then cooked by restaurant staff; these were designated as "self-selected foods." Similar items included simple dormitory-prepared meals. In contrast, "non-self-selected foods"-the predominant type consumed by students-encompassed standard takeout box meals or buffet servings (wherein portion sizes of cooked dishes remained relatively fixed despite individual dish selections). Additionally, accounting for shared eating occasions among participants, both self-selected and non-self-selected foods required documentation of the number of co-diners and their gender distribution.

Third, nutritional information was classified into two categories: hand-made food and pre-prepared meals, which may be partially or completely processed.

“Hand-made food” were dishes prepared from raw ingredients. They were analyzed using Chinese and Japanese food composition tables [23,24] with ingredients and quantities tracked manually. Food quantities were measured for uncooked ingredients.

“Pre-prepared meals” were items that have undergone processing and cooking. Additionally, certain dishes incorporated components from both categories 1 and 2. When available, nutrition composition labels were used for food products and pre-prepared meals. Then, the Chinese and Japanese food composition tables were utilized when combined homemade foods were involved [23,24]. Finally, Chinese food databases such as Booheejiankang [25] were used to find comparable dishes when necessary.

In Chinese cuisine, oil is usually added using a cooking scoop, whether to a heated pan, for deep-frying vegetables, or in soup preparation. Since this method does not allow for exact measurement, the scoop's volume was determined beforehand and converted, following the same approach used for seasonings. Additionally, for cooked foods that require further steps like frying or steaming after packaging, changes in water content and oil weight during each process were based on data from the Japanese database [26]. Finally, after determining the nutritional content of all foods and dishes, the participants' food records were analyzed to assess their intake of energy, carbohydrates, fat, and protein.

The average total energy and macronutrient intakes were calculated, along with snack calorie intake (kcal) and the snack calorie ratio (%). Snack calorie intake is shown as the average daily snack consumption, and the snack calorie ratio is determined by dividing the snack calorie intake by the total calorie intake, expressed as a percentage. Regular meal frequency was determined by summing the total number of meals consumed over three days, resulting in possible scores from 0 to 9. Breakfast consumption and late-night snacking frequencies were each recorded over three days, with possible values from 0 to 3. Participants who consumed these meals each time were compared with those who skipped at least one instance.

Anthropometric measurements

Participants wore T-shirts and shorts without shoes or socks during anthropometric assessments. Height was measured up to the nearest 0.1 cm with a Beryl BYHJ02 stadiometer (Zhongshan Beryl Electronics Co., Ltd., Zhongshan City, China), while weight, BF%, and muscle mass were recorded up to the nearest 0.1 kg with a TANITA BC-314 scale. BMI was determined by dividing weight in kilograms by height in meters squared (kg/m^2^).

Self-administered questionnaire

This study used a questionnaire adapted from a validated survey by Su et al. [27] that assesses health-related behaviors among Chinese university students. It addresses individual characteristics and university lifestyle factors, including sleep patterns, eating habits, physical activity, and food acquisition methods such as online delivery services. Additionally, participants rated their current body shape using a five-point scale: very thin, slightly thin, normal, slightly overweight, and very overweight. They also reported the average daily duration of mobile phone use over the past month. The response options were Less than 1 hour □ > 1-2 hours □ > 2-3 hours □ > 3-4 hours □ > 4-5 hours □ > 5-6 hours □ > 6-7 hours □ More than 7 hours.

Physical activity

Physical activity was assessed using the Chinese short version of the International Physical Activity Questionnaire (IPAQ) [28]. The IPAQ evaluates physical activity and fitness levels. A prior study has utilized the IPAQ to assess processed food consumption among individuals with NWO in Brazil [29]. The questionnaire classifies activities as walking, moderate-intensity, vigorous-intensity, and sedentary time. These categories were analyzed according to metabolic equivalents (METs), duration, and intensity as specified by the protocol. All procedures involving human participants received approval from the Ethics Committee of Kobe University (No. 1035-3). Informed consent was obtained from all participants in a digital format.

Statistical analyses

Statistical analyses were conducted using IBM SPSS Statistics for Windows, Version 25 (Released 2017; IBM Corp., Armonk, New York, United States) and jamovi 2.7.12.0 [30,31]. Continuous variables are reported as means with standard deviations (mean ± SD), while categorical variables are presented as frequencies. The chi-square test was used to assess differences among categorical variables, and independent sample t-tests were used to compare differences among continuous variables, including lifestyle factors and body composition, between university students classified as NWO and non-NWO. A logistic regression model was applied to assess the associations between the independent variables and NWO/non-NWO. In the logistic regression model, body image was transformed to a binary variable using the cutoff value determining normal weight and slightly overweight, while the duration of mobile use was transformed to a binary value using a cutoff value of four hours.

## Results

No male participant was classified as having NWO, whereas 24 female participants were categorized as having NWO. Additionally, 18 male and 46 female participants were classified as non-NWO. The prevalence of NWO was significantly higher among female than among male participants. Although male participants with overweight or obesity were proportionally more prevalent than were female participants with these conditions, only the latter were involved in the subsequent analysis because none of the former met the criteria for NWO.

Participants with NWO (n=24 (34%)) exhibited significantly higher weight, BMI, and BF% than those categorized as non-NWO (n=46 (66%)) (Table 1). Additionally, female participants with NWO perceived their body shape as more obese than the corresponding perceptions of participants in the non-NWO group (Table 1). The NWO group had longer mobile phone usage duration than the non-NWO group (Table 1).

**Table 1:** Comparisons of anthropometric measurements, lifestyle characteristics, and key nutrient intake levels between the groups Comparisons were performed using Welch’s t-test. NWO, normal weight obesity; BMI, body mass index; BF%, body fat percentage.

Factors	NWO (N=24)		Non-NWO (N=46)		t-value	p-value
	Mean	SD	Mean	SD		
Weight (kg)	58.0	4.6	50.3	4.3	6.76	<0.001
BMI	22.6	1.0	19.8	0.9	11.85	<0.001
BF%	32.3	1.3	25.6	2.9	12.94	<0.001
Perception of body build image	3.6	0.5	3.1	0.6	3.69	<0.001
Mobile usage time	7.0	1.3	6.1	1.6	2.57	0.013
Calorie intake (kcal)	1466.0	380.0	1382.6	403.8	0.85	0.398
Protein intake (g)	60.1	18.2	56.2	19.5	0.84	0.404
Fat intake (g)	49.9	17.6	50.9	19.1	−0.21	0.834
Carbohydrate intake (g)	196.1	64.2	181.8	64.1	0.88	0.381
Snack calorie intake (kcal)	107.4	104.7	66.9	89.1	1.62	0.114
Snack-calorie ratio (%)	8.3	8.5	4.5	5.9	1.92	0.063

Nutritional assessment

We identified no statistically significant association between calorie intake (t=0.85, p=0.398, Table 1) or other macronutrient consumption, including protein, fat, and carbohydrates, and NWO status. The average energy intake for the non-NWO and NWO groups was 1,382 (SD=403) kcal and 1,466 (SD=379) kcal, respectively. Protein intake averaged 56 (SD=19.5) g and 60 (SD=18.2) g, fat intake was 51 (SD=19.1) g and 50 (SD=17.6) g, and carbohydrate intake was 182 (SD=64.1) g and 196 (SD=64.2) g for the non-NWO and NWO groups, respectively. However, higher association was observed between snack calorie intake and NWO status (Table 1), as well as for the snack calorie ratio (Table 1). The snack calorie ratio was 4.5% in the non-NWO group and 8.3% in the NWO group. The macronutrient balance was similar between the two groups, with protein, fat, and carbohydrate contributing 16%, 32%, and 52% of total calorie intake in the NWO group, compared to 16%, 31%, and 53% in the non-NWO group, respectively. Dietary habits, such as having regular meals (0.391, p>0.05), eating breakfast (0.225, p>0.05), and late-night eating, were not associated with NWO status (0.723, p>0.05). The results of the logistic regression analysis (Table 2) revealed a statistically significant association between body image and NWO (OR = 8.7, 95%CI = 2.4-31.2).

**Table 2:** Logistic regression analysis of nutrient intakes, body image, and mobile use as predictors of normal weight obesity Models estimated using a sample size of N=70 Binomial logistic regression analysis was used. Estimates represent the log odds of "NWO Logis = NWO" vs. "NOW Logis = Non-NWO" NWO, normal weight obesity.

					Odds ratio		
Predictor	Estimate	SE	Z	p-value	Estimate	95% Confidence Intervals	
Intercept	−3.846	1.487	−2.587	0.010	0.021	0.001	0.394
Protein intake (g)	0.021	0.019	1.068	0.285	1.021	0.983	1.061
Fat intake (g)	−0.025	0.018	−1.374	0.170	0.975	0.940	1.011
Carbohydrate intake (g)	0.007	0.006	1.280	0.201	1.007	0.996	1.019
Snack calorie intake (kcal)	0.004	0.003	1.324	0.186	1.004	0.998	1.011
Body image (normal or thinner to obese)	2.158	0.654	3.298	< .001	8.650	2.400	31.177
Mobile use (≥4 hours or <4 hours)	0.864	0.992	0.871	0.384	2.372	0.339	16.584

Physical assessment

No statistically significant association was identified between METs and any categorized physical activity level or between sedentary time and NWO status (=0.158, p>0.05, Table 3).

**Table 3:** Chi-square test on categorized physical activity levels Comparisons were performed using the chi-square test. NWO, normal weight obesity.

Factors	Activity levels			χ2	p-value
	Low	Moderate	High		
Non-NWO	19	18	9	0.158	0.924
NWO	11	9	4		

## Discussion

This study demonstrated that female university students showed significantly higher prevalence of NWO than did their male counterparts. Previous national research on Chinese adults reported a higher prevalence of NWO in male individuals (10%) than in female individuals (6%) [32]. In contrast, studies focusing on Chinese university students found significantly higher NWO prevalence among female (28%-45%) than among male individuals (14%-26%) [33-35]. Other research revealed NWO prevalence rates of 47% and 18% in Thai and Japanese female students, respectively [18,36]. The results demonstrate that university students, particularly female students, exhibit an increased risk of developing NWO, aligning with the findings of the present study. This relationship likely results from the influence of sex hormones on NWO development [37]. Regarding dietary intake, no significant associations were identified between NWO and total calorie intake. This finding aligns with previous research [14,15]. Notably, both study groups had low energy intake. According to China's dietary reference intakes, the estimated energy requirements are 1800 kcal for women with lower physical activity levels [38]. A previous Japanese study focusing on female university students reported 1855 kcal in NWO groups and 1964 kcal in non-NWO groups [39], while another study involving Japanese female university students reported significantly lower energy intakes of 1240 kcal and 1400 kcal in the NWO and non-NWO groups, respectively [40]. This result closely aligns with our findings. One possible reason for the low energy intake in both groups is that 61% of all participants did not eat breakfast every day, which may lead to not meeting their daily energy needs. No significant association was detected between NWO status and macronutrient (protein, carbohydrates, and fat) intake. Our participants met the estimated energy requirement thresholds for protein (50 g) and carbohydrate (120 g) [38]. Fat intake exceeded 30% in both groups, which is considered relatively high compared to the recommended levels [38,41].

Notably, although a statistically significant association was not observed, individuals with NWO had higher snack-calorie intake than those without NWO. Snacking refers to the consumption of any food or beverages outside of main meals, specifically breakfast, lunch, and dinner. These instances are typically classified as morning, afternoon, and night snacks and are collectively referred to as “snack occasions" [42]. Unhealthy snack consumption contributes to increased BF% and NWO prevalence. In Indonesia, half of the university students surveyed recognized the impact of unhealthy snacks on BMI and BF% [43]. Additionally, women aged <45 years who frequently consumed unhealthy snacks had higher BF% and increased NWO prevalence [44]. Previous research on snack composition demonstrates that university students with NWO consume a higher proportion of unhealthy snacks than do non-NWO peers [18]. Meanwhile, the present study found a tendency toward higher snack-calorie intake in individuals with NWO; however, the snack-calorie ratio (%) was relatively lower than in previous studies. For example, young adults in the U.S. derive approximately 20% of their daily energy from snacks [45], while the corresponding values for schoolchildren in the Philippines and Russia are 18% and 15%, respectively [46]. Among Chinese adults, the snack-calorie ratio (%) ranges from 4.1% to 12.3% [42], consistent with our study findings. Future research should further investigate snack intake among Chinese female university students. Additionally, regarding dietary habits, a study focusing on 18-year-olds in Iceland reported a difference in daily breakfast frequency between NWO and non-NWO groups [15]. However, our study did not find a link between the study groups, which aligns with a survey of Thai female college students [18]. Additionally, the difference in late-night snacking was not observed in our study, similar to other studies [15,18].

No statistically significant association was identified between NWO and overall physical activity, including sedentary time. Previous studies have examined physical fitness levels among individuals with NWO and those categorized as non-NWO. One study of Chinese university students assessed activities such as jumping and running, revealing that individuals with NWO exhibited lower physical fitness levels [35]. Additionally, studies using questionnaires with Chinese university students indicated poor physical activity-related behaviors [33,34]. Research involving adults in the United States found that both the NWO and overweight-obesity groups had lower fitness levels than did individuals without NWO [16]. Meanwhile, individuals with an elevated waist circumference ratio demonstrated reduced physical strength [47]. Among the possible reasons that the physical assessment results in this study did not match those of previous research is that many young people in China tend to have low levels of physical activity and a high amount of sedentary time [48]. Consequently, detecting differences may require larger samples. Additionally, variations in measurement scales for physical activity levels, or inconsistent cut-off criteria for NWO, can affect study outcomes [33-35]. Furthermore, long-term smartphone usage may indirectly contribute to obesity by increasing sedentary time [49]. However, the relationship between mobile phone usage and NWO status has not been investigated, and further research is required.

The college period is a critical stage for the development of healthy behaviors, offering students opportunities to enhance dietary patterns and lifestyle practices. In addition, implementing obesity prevention strategies is vital for reducing disease prevalence among university populations. Overweight and obese college students reportedly experience high levels of dissatisfaction with their weight [50] and often underestimate their body weight [51]. Also, their quality-of-life scores are consistently lower than those of individuals with normal weight [52,53]. The present study is the first to compare dietary intake between individuals with NWO and those without NWO among Chinese female university students, utilizing dietary records. These results tended to indicate a difference in snack-calorie intake between the NWO and non-NWO groups, rather than regarding the total energy or macronutrient intake. This suggests that snack intake may be a contributing factor to being NWO. This finding may inform novel prevention and intervention programs targeted at female college students with NWO.

This study has several limitations. First, the use of convenience sampling likely introduced participant selection bias, which restricts the generalizability of the findings. Second, the cross-sectional design precludes the determination of causal relationships. Third, the relatively small sample size (NWO and non-NWO = 24 and 46, respectively) may have limited the statistical power of multivariate analyses adjusted for potential confounders. Although the initial study design incorporated additional variables, the effective sample size reduced due to missing data. Furthermore, the stratified analysis did not yield statistically significant findings, which is likely attributable to the low statistical power resulting from the small sample size due to unexpected missing fields in the questionnaires completed by some participants. Finally, additional factors that may influence the development of NWO in students, including sleep patterns, dietary habits, genetic predispositions, and stress levels, were not addressed.

## Conclusions

Individuals with NWO tended to have higher snack-calorie intake than non-NWO individuals. No association was identified between total calorie intake, macronutrient intake, or physical activity levels. These results provide evidence to inform lifestyle interventions targeting NWO. Further large-scale studies examining snack consumption volume and patterns are required to validate these findings.

## Appendices

**Table 4:** Study questionnaire (English version) Questionnaire adapted from ref [27,28] Ref [27] is an open access article, distributed under the terms of the Creative Commons Attribution license (http://creativecommons.org/licenses/by/4.0/), which permits unrestricted re-use, distribution and reproduction The IPAQ [28] is in the Public Domain.

Questionnaire Incorporating the Chinese short version of the International Physical Activity Questionnaire											
Registration number	Date of interview										
1	Demographic profile										
1.1	Gender	Male	Female								
1.2	Ethnicity	Han	Minority								
1.3	Birthday	year	month	date							
1.4	Residence	Province	City								
1.5	Raised place	Province	City								
1.6	What are your monthly living expenses?	<1000 yuan	1001-1500 yuan	1501-2000 yuan	>2001 yuan						
1.7	On average, how much money do you spend on food within a month?	yuan									
2	Family										
2.1	Are you an only child?	Yes	No								
2.2	What is your family type?	Two-parent family (both biological parents)	Two-parent family (1 biological parent and 1 step-parent)	Single-parent family	Other						
2.3	How do you think your family’s financial conditions are subjectively?	Extreme poor	Poor	Moderate	Affluent	Extreme affluent					
2.4	Father’s education	Primary school or below	Middle school	High school/secondary	College education or more						
2.5	Mother’s education	Primary school or below	Middle school	High school/secondary	College education or more						
3	University										
3.1	Campus name	Guangzhou	Sanshui								
3.2	School year	1st year	2nd year	3rd year	4th year	more than 4th year					
3.3	Grade	Undergraduate	Graduated								
3.4	Faculty	School									
3.5	Where is your living place while studying?	Home	Student accommodation on campus	Non-student accommodation on campus	Outside apartments						
3.6	Do you have a job while studying?	Yes	No								
4	Health										
4.1	What do you generally consider your health condition?	Very good	Relatively good	Fair	Relatively poor	Very poor					
4.2	What do you consider your current health status compared with 1 year ago?	Much better than 1 year ago	Better than 1 year ago	About the same as 1 year ago							
4.3	How do you think your body type belongs to	Very thin	Thin	Medium	Fat	Very fat					
4.4	Do you currently have dietary restrictions?	Currently on a diet	I have done in the past	Never have done							
4.5	Do you pay attention to your health?	Very concerned	Somewhat concerned	Neither	Not very concerned	Not concerned at all					
4.6	Do you care about your health	Care very much	Somewhat care	Neither	Do not care much	Do not care at all					
4.7	How is the nutritional balance of your daily diet?	Very good	Fairly good	Neither	Slightly poor	Poor					
4.8	What is your understanding of diet?	I consciously choose to eat foods that are beneficial to health	Do not pay much attention	It’s fine as long as it tastes good	Other						
5	Lifestyle										
5.1	What time do you usually sleep in the last month?	am/pm									
5.2	What time do you usually get up in the last month?	am/pm									
5.3	Do you have the habit of taking a nap?	Yes, how long?	No								
5.4	How do you feel about your sleeping quality in the last month?	Very good	Good	Fair	Not good	Very bad					
5.5	Do you feel stressed?	Not really	Occasionally	Often							
5.6	Do you feel fatigued?	Not really fatigued	Occasionally feel fatigued	Often feel fatigued							
5.7	In the past month, how long did you spend using your phone for internet access each day?	Less than 1 hour	> 1–2 hours	> 2–3 hours	> 3–4 hours	> 4–5 hours	> 5–6 hours	> 6–7 hours	More than 7 hours		
5.8	What are your main purposes for using it? (Multiple answers allowed)	Communication (SNS, app calls, etc)	Gaming	Watching/posting short or long videos	Browsing and commenting on blogs, forums, or websites	Online courses or software learning	Reading e-books or comics	Information searching	Online shopping or auctions	Other	
5.9	How often did you smoke cigarettes in the last month?	Not smoking	Once a week 2 to 3 days a week	4 to 5 days a week	Almost every day						
5.10	How often did you drink alcohol in the last month? (A portion of alcohol is equivalent to half of a bottle/a can of beer (350- 600 ml), a glass of Western white/red wine(200ml), a cup of rice wine(250ml), not just a sip)	Not drinking	Once a week	2 to 3 days a week	4 to 5 days a week	Almost every day					
5.11	How many days per week and how long are your physical education classes?	0 day	1 day	2 days	3 days	4 days	5 days or more				
6	Physical activity										
6.1	How many days did you do vigorous physical activities during the last 7 days? Vigorous physical activities refer to making hard physical effort for at least 10 minutes at a time, and it makes you breathe much harder than normal. (For example: Vigorous activities refer to running, climbing uphill, continuously fast swimming (excluding slow swimming, water playing), aerobic dance, fast cycling, playing ball games (such as tennis, basketball, soccer, badminton, table tennis), weight training, continuously rope skipping.	days per week	No vigorous physical activities								
6.2	How much time did you usually spend on doing vigorous physical activities on one of those days?	hours	minutes per day	Don’t know/Not sure							
6.3	How many days did you do moderate physical activities during the last 7 days? Moderate activities refer to making moderate physical effort for at least 10 minutes at a time, and it makes you breathe somewhat harder than normal. (For example: Swimming at normal speed, going down the stairs, slow dancing(excluding street dance and martial arts), riding a bicycle at normal speed, laborious housework (washing windows, wiping the floor by hand, making the bed, washing clothes by hand), please don't count the light-weight walking.	days per week	No moderate physical activities								
6.4	How much time do you usually spend doing moderate physical activities on one of those days?	hours	minutes per day	Don’t know/Not sure							
6.5	How many days did you walk for at least 10 minutes at a time during the last 7 days? (This includes walking from place to place at work and at home, and any other walk that you have done solely for recreation, sport, exercise, or leisure)	days per week	No walking								
6.6	How much time do you usually spend walking on one of those days?	hours	minutes per day	Don’t know/Not sure							
6.7	How much time did you spend sitting during the last 7 days? (This includes sitting down for studying, using a computer/mobile phone at school or other places, sitting while chatting with friends, lying down on the couch while watching television or using a mobile phone. But please don't count the time you fall asleep)	hours	minutes per day	Don’t know/Not sure							
Online Food Delivery Service: Online Food Delivery Service (OFDS) in this survey refers to an online application that, on behalf of restaurants, provides food ordering, payment transaction service, and distributing the delivery tasks to individual drivers who deliver food to customers.											
7	Dietary pattern										
7.1	In the past 7 days, approximately how many times did you have breakfast? Please check the frequency records of your food delivery (ODS) on your mobile phone. (If you had breakfast every day, write 7; if you did not have breakfast at all, write 0.)	times/week	Dine-in at the school cafeteria times +	Dine-in outside the school cafeteria times +	Takeaway times +	Food delivery times +	Other (e.g., cooking in the dormitory, online shopping) times				
7.2	Did you have breakfast at a fixed time?	Yes, around AM/PM	No fixed time								
7.3	In the past 7 days, approximately how many times did you have lunch? Please check the frequency records of your food delivery (ODS) on your mobile phone. (If you had lunch every day, write 7; if you did not have lunch at all, write 0.)	times/week	Dine-in at the school cafeteria times +	Dine-in outside the school cafeteria times +	Takeaway times +	Food delivery times +	Other (e.g., cooking in the dormitory, online shopping) times				
7.4	Did you have lunch at a fixed time?	Yes, around AM/PM	No fixed time								
7.5	In the past 7 days, approximately how many times did you have dinner? Please check the frequency records of your food delivery (ODS) on your mobile phone. (If you had dinner every day, write 7; if you did not have dinner at all, write 0.)	times/week	Dine-in at the school cafeteria times +	Dine-in outside the school cafeteria times +	Takeaway times +	Food delivery times +	Other (e.g., cooking in the dormitory, online shopping) times				
7.6	Did you have dinner at a fixed time?	Yes, around AM/PM	No fixed time								
7.7	Among breakfast, lunch, and dinner, if you skipped one or more meals, what were the reasons? (Multiple answers allowed)	I did not have time to eat	I had no appetite	I am not in the habit of eating regularly	It is inconvenient to go to the cafeteria	It is inconvenient to have breakfast	I am dieting (trying to lose weight)	To save money	Other		
7.8	In the past 7 days, approximately how many times did you have a snack (food between meals)? Please check the frequency records of your food delivery (ODS) on your mobile phone. (If you had a snack every day, write 7; if you did not have a snack at all, write 0.)	times/week	Dine-in at the school cafeteria times +	Dine-in outside the school cafeteria times +	Takeaway times +	Food delivery times +	Other (e.g., cooking in the dormitory, online shopping) times				
	Among these, approximately how many times did you have snacks after 9:00 PM?	times/week	Dine-in at the school cafeteria times +	Dine-in outside the school cafeteria times +	Takeaway times +	Food delivery times +	Other (e.g., cooking in the dormitory, online shopping) times				
7.9	What do you think are the benefits of eating snacks? (Multiple answers)	Mood improvement	Satisfying hunger	Nutritional supplementation	Replacing meals	Habit	Other				
7.10	What is your main motivation for using online food delivery service? (Multiple answers)	Saving time (due to busy schedule)	Saving time (due to physical demand of going to eat at canteen or restaurant)	Better taste	Better quality	Food only available through ordering online app in certain period of time	Cheaper price	Avoiding bad weather	No peers to eat together	Other	
7.11	Overall, are you satisfied with online food delivery service?	Very satisfied	Satisfied	Neutral	Not satisfied	Not satisfied at all					
